# Development of recombinant COVID-19 vaccine based on CHO-produced, prefusion spike trimer and alum/CpG adjuvants

**DOI:** 10.1016/j.vaccine.2021.10.066

**Published:** 2021-11-26

**Authors:** Haitao Liu, Chenliang Zhou, Jiao An, Yujiao Song, Pin Yu, Jiadai Li, Chenjian Gu, Dongdong Hu, Yuanxiang Jiang, Lingli Zhang, Chuanqi Huang, Chao Zhang, Yunqi Yang, Qianjun Zhu, Dekui Wang, Yuqiang Liu, Chenyang Miao, Xiayao Cao, Longfei Ding, Yuanfei Zhu, Hua Zhu, Linlin Bao, Lingyun Zhou, Huan Yan, Jiang Fan, Jianqing Xu, Zhongyu Hu, Youhua Xie, Jiangning Liu, Ge Liu

**Affiliations:** a Shanghai Zerun Biotechnology Co., Ltd., Shanghai, China; b Institute of Laboratory Animal Sciences, Chinese Academy of Medical Sciences and Comparative Medicine Center, Peking Union Medical College, Beijing, China; c Key Laboratory of Medical Molecular Virology (MOE/NHC/CAMS), Department of Medical Microbiology and Parasitology, School of Basic Medical Sciences, Shanghai Medical College, Fudan University, Shanghai, China; d National Institute for Food and Drug Control (NIFDC), Beijing, China; e Shanghai Public Health Clinical Center, Fudan University, Shanghai 201508, China; f State Key Laboratory of Virology, Modern Virology Research Center, College of Life Sciences, Wuhan University, Wuhan, China

**Keywords:** SARS-CoV-2, Trimeric spike protein, Subunit vaccine

## Abstract

COVID-19 pandemic has severely impacted the public health and social economy worldwide. A safe, effective, and affordable vaccine against SARS-CoV-2 infections/diseases is urgently needed. We have been developing a recombinant vaccine based on a prefusion-stabilized spike trimer of SARS-CoV-2 and formulated with aluminium hydroxide and CpG 7909. The spike protein was expressed in Chinese hamster ovary (CHO) cells, purified, and prepared as a stable formulation with the dual adjuvant. Immunogenicity studies showed that candidate vaccines elicited robust neutralizing antibody responses and substantial CD4^+^ T cell responses in both mice and non-human primates. And vaccine-induced neutralizing antibodies persisted at high level for at least 6 months. Challenge studies demonstrated that candidate vaccine reduced the viral loads and inflammation in the lungs of SARS-CoV-2 infected golden Syrian hamsters significantly. In addition, the vaccine-induced antibodies showed cross-neutralization activity against B.1.1.7 and B.1.351 variants. These data suggest candidate vaccine is efficacious in preventing SARS-CoV-2 infections and associated pneumonia, thereby justifying ongoing phase I/II clinical studies in China (NCT04982068 and NCT04990544).

## 1 Introduction

The pandemic of coronavirus disease 2019 (COVID-19), which is caused by severe acute respiratory syndrome coronavirus 2 (SARS-CoV-2) [1], has severely impacted the public health and global economy. Since the first cases of COVID-19 were reported in December 2019 [2], numerous researchers have taken great efforts to control this disease. Development of prophylactic vaccines against SARS-CoV-2 is a proven strategy to prevent and to terminate the unprecedented pandemic [3]. Currently, different types of vaccines have been developed or under development against SARS-CoV-2 [4]. Several of them have completed Phase III clinical trials and demonstrated to be efficacious in preventing SARS-CoV-2 infections and/or reducing the occurrence of severe symptoms, hospitalization rate, and death caused by SARS-CoV-2 infections. As the demand for SARS-CoV-2 vaccines is huge, current approved SARS-CoV-2 vaccines cannot meet the requirement of removing COVID-19 pandemic with rapidity. In addition, vaccinees may choose different type of vaccines according to the age, health status and affordability. Thus, it is necessary to develop SARS-CoV-2 vaccines with different platforms. Here we report the development of a modified prototype spike protein-based vaccine combined with Alum/CpG dual adjuvant system.

SARS-CoV-2 invades into host cells by engaging the receptor binding domain (RBD) of spike glycoprotein with angiotensin-converting enzyme 2 (ACE2) on host cell surface [5]. Based on the cell entry mechanism, spike glycoprotein is a reasonable vaccine target. In line with this hypothesis, it was confirmed that plenty of neutralizing antibody (nAb) epitopes reside in spike glycoprotein [6], [7]. Spike glycoproteins are displayed on the exterior of SARS-CoV-2 virion as a trimer. We hypothesized that spike trimer in the prefusion conformation is highly antigenic, a lesson learned from the vaccine development targeting respiratory syncytial virus (RSV), MERS, and SARS [8], [9]. Therefore, we designed a prefusion-stabilized spike trimer as the vaccine target, named SΔTM. Chinese hamster ovary (CHO) cell expression system was used to express the target antigen, as it has sophisticated glycosylation system, which may be essential to SΔTM’s immunogenicity.

To elicit maximum immune responses, we incorporated a dual adjuvant system into our candidate vaccine, which contains aluminium hydroxide (Alum) and CpG 7909 (CpG). Aluminium salts have been used in vaccines for approximately 100 years with an excellent record of safety and effectiveness. Though the mechanisms of action of alumunium adjuvants are controversial, it is demonstrated that they can help induce T helper type 2 (Th2) cell-associated antibody responses [10]. In addition, aluminium salts are able to absorb and stabilize antigens formulated in vaccines [11], which contributes to the stability of vaccine immunogens and benefits the process of vaccine production. CpG 7909 is a synthetic oligonucleotide, a ligand of Toll-like receptor 9 (TLR9) [12]. By binding to endogenous TLR9 in B cells, dendritic cells (DCs), or macrophages, CpG 7909 activates MyD88 signal pathway and induce proinflammatory immune responses [12], [13]. In addition, CpG 7909 activates DC to upregulate costimulatory molecules and activation markers to promote their homing to draining lymph nodes [14]. As a result, CpG 7909 help organisms to induce Th1-biased cellular and humoral immune responses, which confer the protection against infection. As the dual adjuvant system possesses the advantages of both aluminium salts and CpG oligonucleotide, vaccine targets adjuvanted with this adjuvant system are likely to induce high level of antibody responses associated with Th1-biased immunity. In terms of development of SARS-CoV-2 vaccine, Th1-biased immune responses may reduce the potential of vaccine-enhanced diseases (VED) [15], [16], [17], though no VED was reported in completed clinical trials and post clinical trial studies so far.

In this study, we report the excellent antigenicity of immunogens, immunogenicity of the vaccine candidate in rodents and nonhuman primate (NHP) models as well as the efficacy of the vaccine candidate in a hamster challenge study. We also analyzed the cross-neutralizing activity of the immune sera of NHP. The results suggest our vaccine candidates are promising and support further clinical development.

## 2 Results

### 2.1 Immunogen design and characterization

**Fig. 1:**
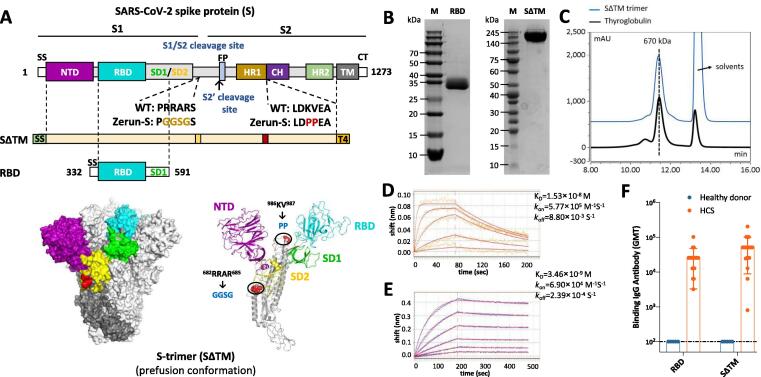
**Molecular design and characterization of SΔTM and RBD.** (**A**) Domain architecture of the SARS-CoV-2 S protein. SS, signal sequence; NTD, N-terminal domain; RBD, receptor-binding domain; SD1, subdomain 1; SD2, subdomain 2; S1/S2, S1/S2 protease cleavage site; S2′, S2′ protease cleavage site; FP, fusion peptide; HR1, heptad repeat 1; CH, central helix; CD, connector domain; HR2, heptad repeat 2; TM, transmembrane domain; CT, cytoplasmic tail. Two recombinant SARS-CoV-2 spike antigens were designed: SΔTM (the prefusion S ectodomain with proline substitutions at residues 986 and 987 to retain S2 in the prefusion conformation, a “GGSG” substitution at the furin cleavage site, a C-terminal T4 fibritin trimerization motif), and 260-mer RBD (RBD-SD1). The structure model of S-trimer was generated by the SWISS-MODEL using homology modelling techniques (http://swissmodel.expasy.org/), and the 3D structure figures were prepared using PyMOL (www.pymol.org). (**B**) SDS-PAGE analysis of purified SΔTM and RBD. Molecular weight standards are indicated at the left in kDa. (**C**) Size-Exclusion HPLC chromatogram of purified SΔTM (shown as blue line) and a 670 kDa molecular weight standard (shown as black line). (**D**) and (**E**) Binding profiles of SΔTM and RBD to human ACE2 measured by BLI in GatorPrime. The data are shown as blue and orange lines for SΔTM and RBD, respectively, and the best fit of the data to a 1:1 binding model is shown in red. (**F**) Antigenicity of SΔTM and RBD measured by serially diluted HCS. HCS, human convalescent sera.

As it is the initial binding site of SARS-CoV-2 during its invasion into host cells by interacting with ACE2, spike protein is an ideal choice of vaccine immunogen. Here we designed both SΔTM and RBD as vaccine components shown in Fig. 1A. SΔTM derives from ectodomain of spike protein with two mutation sites and conjugates with a T4 fibritin trimerization motif, whereas monomer RBD is a part of S1 domain, including RBD region and SD1 region. Both recombinant proteins were expressed by CHO cells, and purified by multi-step chromatography. The purified proteins were characterized by SDS-PAGE, size-exclusion chromatography (SEC), and biolayer interferometry (BLI). SDS-PAGE analysis showed that the purity of both SΔTM and RBD are over 90% (Fig. 1B). The SEC results showed that retention time of purified SΔTM was similar with a 670 kDa standard protein, thyroglobulin, indicating the SΔTM was in its trimeric form (Fig. 1C). The binding affinity of RBD and SΔTM to human ACE2 was determined by BLI, with dissociation constant (K_D_) of 1.53 × 10^-8^ M and 3.46 × 10^-9^ M, respectively (Fig. 1D and 1E). In addition, both RBD and SΔTM were recognized by highly diluted human convalescent sera (HCS) (Fig. 1F), indicating their good antigenicity.

### 2.2 Immunogenicity of candidate vaccine in mice

**Fig. 2:**
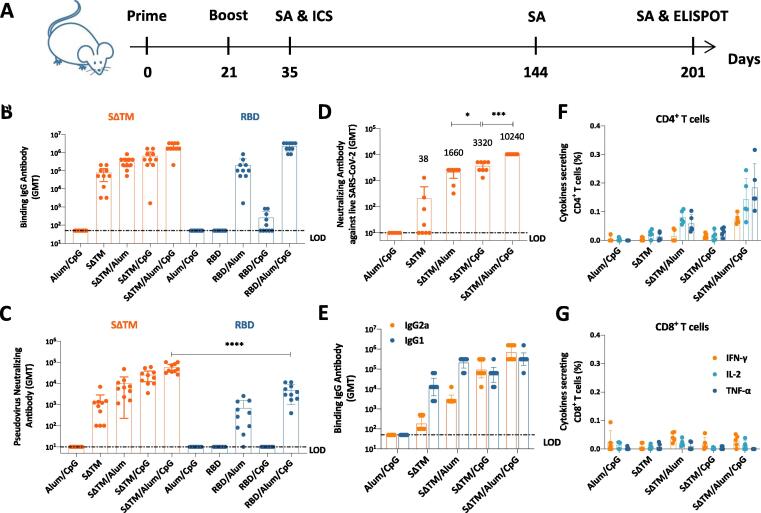
**Immune responses in vaccinated BALB/c mice**. (**A**) Experiment schedule. BALB/c mice were immunized twice intramuscularly at Day 0 and Day 21. Vaccine components were 5 μg SΔTM or RBD, which adjuvanted with 50 μg Alum and/or 50 μg CpG. Alum/CpG only groups were as control. On Day 35, Day 144 (only SΔTM groups) and Day 201 (only SΔTM groups), blood was collected to perform serological assays (SAs) (N = 10). On Day 35, 5 mice from each SΔTM groups were sacrificed to conduct intracellular staining (ICS) assay (N = 5). On Day 201, 5 mice from each SΔTM groups were sacrificed to conduct B cell ELISPOT (N = 5). (**B**) Binding antibody titers and (**C**) pseudovirus neutralizing antibody titers of sera collected at Day 35 from SΔTM or RBD vaccinated mice. (**D**) Neutralizing antibody titers of sera collected at Day 35 from SΔTM vaccinated mice detected by live virus-based neutralization assay. (**E**) IgG isotyping of sera collected at Day 35 from SΔTM vaccinated mice. (**F**) Antigen specific CD4^+^ T cell responses and (**G**) CD8^+^ T cell responses in SΔTM vaccinated mice were determined by ICS at Day 35. Dotted lines represent the limit of detection (LOD). Each dot represents an individual mouse. Numbers on the top of each bar represent geometric mean titers. Statistical analysis was performed using *t*-test with Welch’s correction. *P < 0.05; ****P < 0.0001.

Purified subunit proteins alone are poorly immunogenic as they are lack of immunostimulatory capacity. Adjuvants are capable of enhancing vaccine-induced adaptive immune responses by triggering innate immune responses. In addition, as it was reported nAb epitopes mainly locate in the RBD region of spike protein, we expected to compare the immunogenicity of SΔTM and RBD. We performed a mouse study to select the best combination of antigen and adjuvant. In this study, BALB/c mice were immunized intramuscularly twice at a 3-week interval with 5 μg SΔTM or RBD, formulated with Alum, CpG or Alum/CpG (Fig. 2A). Binding antibody titers and nAb titers were detected by ELISA and pseudovirus-based neutralization assay 2 weeks post second immunization, respectively (Fig. 2B and 2C). Results showed that all SΔTM groups induced high titers of anti-RBD antibody with geometric mean titers (GMT) ranging from 3.9 × 10^4^ to 1.8 × 10^6^. In contrast, RBD adjuvanted with Alum alone or Alum/CpG induced high titers of anti-RBD antibody, 9.6 × 10^4^ (GMT) and 2.0 × 10^6^ (GMT), respectively. In consistent with ELISA results, all adjuvanted SΔTM groups induced high titers of nAbs with GMT ranging from 624 to 51939. SΔTM alone without adjuvant showed relatively poor immunogenicity. Unexpectedly, all RBD groups elicited weak nAb responses with the highest nAb titers of 3429 (GMT) in Alum/CpG adjuvanted group. These results indicate SΔTM as vaccine component has advantage over RBD in terms of immunogenicity.

We then detected vaccine-induced nAb responses in SΔTM groups via live virus-based neutralization assay (Fig. 2D). SΔTM alone without adjuvant elicited weak neutralizing activity. By contrast, SΔTM adjuvanted with either Alum, CpG or Alum/CpG elicited high titers (GMT) of nAbs, ranging from 1660 to 10240. In detail, the GMTs of nAb induced by SΔTM/Alum/CpG group were 3-fold and 6-fold of those by SΔTM/CpG group and SΔTM/Alum group, respectively. Next, we determined the IgG subclasses of sera from SΔTM groups (Fig. 2E). Results showed the ratio of IgG2a/IgG1 in SΔTM/CpG group or SΔTM/Alum/CpG was reversed compared with SΔTM alone group or SΔTM/Alum group, which suggest CpG is able to divert SΔTM induced humoral immune responses into Th1-associated.

Vaccine-induced cellular immune responses are essential to facilitate the production of high-quality antibodies and kill pathogen-invaded host cells. We sacrificed 5 mice in each SΔTM groups to evaluate T cell responses 2 weeks post second immunization (Fig. 2F and 2G). Compared with Alum/CpG group, neither SΔTM alone group nor SΔTM/CpG group elicited antigen-specific CD4^+^ T cell responses; whereas SΔTM/Alum group and SΔTM/Alum/CpG group did, though the responses in SΔTM/Alum group were weak. In this study, we rarely detected CD8^+^ T cell responses in Alum adjuvanted groups.

**Fig. 3:**
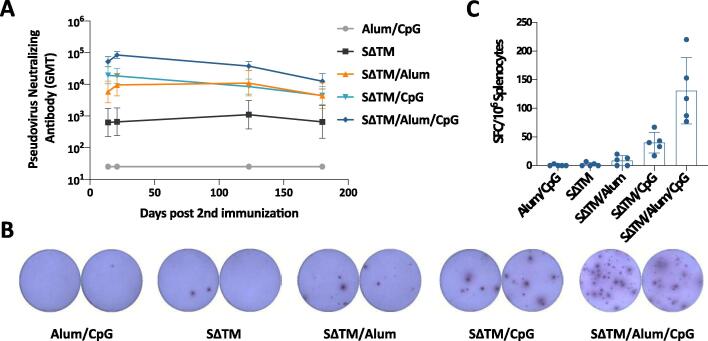
**Immune persistence in vaccinated BALB/c mice.** (**A**) Pseudovirus neutralizing antibody responses monitored at different timepoints in mice vaccinated with different vaccine components as indicated. (**B**) Representative results of SΔTM-specific B cell ELISPOT. (**C**) SΔTM-specific memory B cells detected in each vaccinated group by ELISPOT 180 days post second immunization.

We then monitored the persistence of vaccine-induced immune responses, as this characteristic determines the quality of candidate vaccine in long term protection against SARS-CoV-2. We measured the nAb titers of vaccinated mouse sera from SΔTM groups at different timepoints by pseudovirus-based neutralization assay (Fig. 3A). Results showed that nAb responses peaked at 3 weeks post second immunization and declined thereafter. However, the nAb titers in SΔTM/Alum/CpG group maintained at 12,380 (GMT) even 6 months post second immunization, which indicates that vaccine-induced immune responses are with strong magnitude and good longevity in mice. Subsequently, we sacrificed 5 mice from each group and detected the SΔTM-specific memory B cells 6 months post second immunization (Fig. 3B and 3C). In accordance with data from nAb responses, mice in SΔTM/Alum/CpG group maintained the highest number of SΔTM-specific memory B cells in the spleen, which produce nAbs against SARS-CoV-2.

In summary, SΔTM combined with adjuvants elicited higher titers of nAbs than that elicited by adjuvanted RBD. Furthermore, SΔTM formulated in Alum/CpG adjuvants induced significantly higher levels of both antibody responses and T cell responses than those with either Alum or CpG alone. This result is consistent with previous report [18] and supports the use of dual adjuvants. In addition, candidate vaccines, especially SΔTM formulated in Alum/CpG adjuvants, showed strong and long-lasting immune responses.

### 2.3 Immunogenicity of candidate vaccine in nonhuman primates

**Fig. 4:**
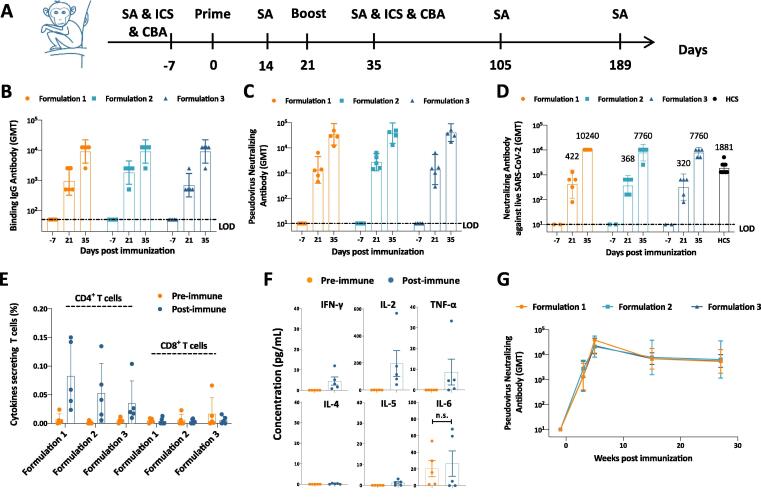
**Immune responses in vaccinated cynomolgus monkeys**. (**A**) Experiment schedule. Each group of Cynomolgus monkey (N-5) were immunized twice intramuscularly with different formulations of vaccines at Day 0 and Day 21. Formulation 1 contained 50 μg SΔTM, 500 μg Alum and 500 μg CpG; Formulation 2 contained 25 μg SΔTM, 500 μg Alum and 500 μg CpG; and Formulation 3 contained 50 μg SΔTM, 500 μg Alum and 250 μg CpG. 7 days before first immunization (Day −7), blood was collected from all individual monkeys to detect the baseline of both humoral and cellular immune responses. Blood was also collected at Day 14, Day 35, Day 105 and Day 189 to perform serological assays (SAs). In addition, PBMCs were isolated at Day 35 to detect cellular immune responses by intracellular cytokine staining (ICS) and cytometric bead array (CBA). (**B**) Binding antibody titers, (**C**) pseudovirus neutralizing antibody titers, and (**D**) live virus neutralizing antibody titers were evaluated at different timepoints as indicated. (**E**) Antigen specific T cell responses in vaccinated monkeys were determined by ICS at Day −7 (Pre-immune, as negative control) and Day 35 (Post-immune). (**F**) Th1-associated cytokines (IFN-γ, IL-2, TNF-α) and Th2-associated cytokines (IL-4, IL-5, IL-6) secreted from formulation 1 vaccine vaccinated monkeys were detected by CBA. (**G**) Pseudovirus neutralizing antibody responses were detected at Day −7, Day 14, Day 35, Day 105 and Day 189 to monitor the immune persistence. Dotted lines represent the limit of detection (LOD). Each dot represents an individual monkey. Numbers on the top of each bar represent geometric mean titers. Statistical analysis was performed using *t*-test with Welch’s correction. n.s.: no significance.

As the immune system of nonhuman primate is closer to that of human being, we next evaluated candidate vaccines with three different formulations in cynomolgus monkeys (Fig. 4A). Formulation 1 contains 50 μg SΔTM/500 μg Alum/500 μg CpG, Formulation 2 contains 25 μg SΔTM/500 μg Alum/500 μg CpG, and Formulation 3 contains 50 μg SΔTM/500 μg Alum/250 μg CpG**.** Each formulation has a volume of 500 μL. In this study, each group of 5 cynomolgus monkeys were immunized twice at a 3-week interval with these 3 formulations. Blood was collected from individual monkeys 1 week ahead of immunization, 3 weeks, 5 weeks, 15 weeks and 27 weeks post immunization. Binding antibody and nAb titers were detected by ELISA and neutralization assays, respectively, at different timepoints (Fig. 4B-D). Results showed that all three formulations induced high titers of both binding and neutralizing antibodies 3 weeks post first immunization. Two weeks after the second shot, the antibody responses became much stronger, ranging from 6566 (GMT) to 9060 (GMT) of binding antibody titers, from 7760 (GMT) to 10,240 (GMT) of live virus-based nAb titers. However, there was no significant difference among these three formulations in antibody responses. As a reference point, the live virus-based nAb titers of human convalescent sera (HCS) were 1881 (GMT).

We then evaluated the cellular immune responses in vaccinated NHPs. PBMCs were separated from whole blood of individual monkeys, which was collected one week before the first immunization and two weeks post the second immunization. SΔTM specific T cell responses were detected by intracellular cytokine staining assay (Fig. 4E). Results showed that all three formulations induced significant CD4^+^ T cell responses after immunization. However, there was no SΔTM-specific CD8^+^ T cells elicited, which is in accordance with the results in mouse study. At the beginning of development of SARS-CoV-2 vaccine, vaccine-enhanced disease was concerned as a potential risk, which was suggested to be related with vaccine-induced Th2-associated responses. Here, we tested the secretion of Th1-associated cytokines (IFN-γ, IL-2, TNF-α) and Th2-associated cytokines (IL-4, IL-5, IL-6) from stimulated PBMC supernatants (Fig. 4F). Results showed that stimulated PBMC mainly secret IFN-γ, IL-2 and TNF-α. No Th2 associated IL-4 or IL-5 was detected. Though the secretion of IL-6 in supernatants were detected, there was no significant difference between samples processed pre- and post-immunization. We suggested the origin of IL-6 was from innate immune cells activated by unknown factors. These results demonstrated the vaccine candidates induce only Th1-associated T cell responses.

We also monitored the persistence of vaccine-induced immune responses by pseudovirus-based neutralization assay in NHPs (Fig. 4G). Compared with the nAb responses 2 weeks post second immunization, the nAb titers (GMT) in each group declined rapidly 12 weeks post second immunization, ranging from 6859 to 7744. However, the nAb titers (GMT) only declined slightly at 24 weeks post second immunization, ranging from 5379 to 6382, compared with that at 12 weeks post second immunization. The results indicate that our candidate vaccines are able to induce persistent nAb responses in NHPs.

### 2.4 Immunogenicity and protective efficacy of candidate vaccines in SARS-CoV-2/hamster model

**Fig. 5:**
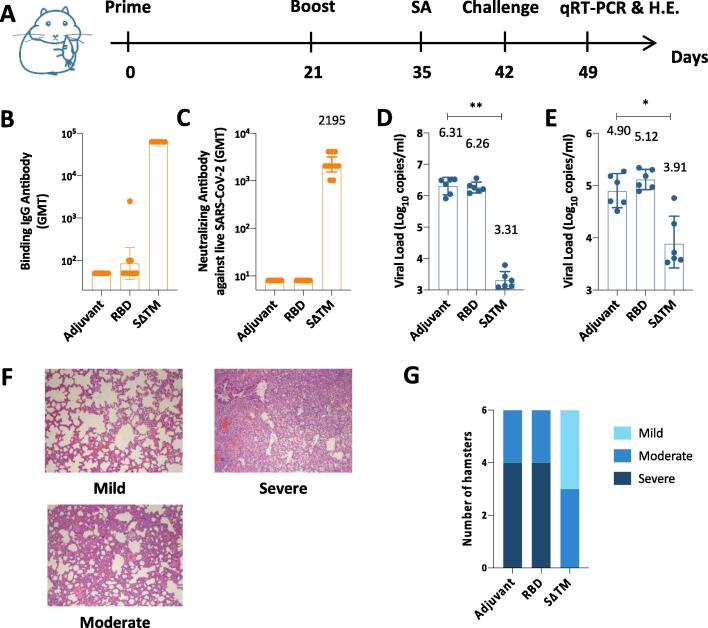
**Immune responses and protective efficacy in vaccinated golden Syrian hamsters**. (**A**) Experiment schedule. Hamsters from different groups (N = 10) were prime-boost immunized intramuscularly at Day 0 and Day 21. Adjuvant group vaccine contained 100 μg Alum and 100 μg CpG; RBD group contained 10 μg RBD, 100 μg Alum and 100 μg CpG, and SΔTM group contained 10 μg SΔTM, 100 μg Alum and 100 μg CpG. Blood was collected at Day 35 from each group to detect antibody responses. On Day 42, all hamsters were challenged intranasally with 10^5^ TCID_50_ SARS-CoV-2. On Day 49, a subset of hamsters in each group (N = 6) was euthanized for detecting viral loads of lungs and nasal turbinates by qRT-PCR and evaluating lung histopathology by hematoxylin and eosin (H.E.) staining. (**B**) Binding antibody titers and (**C**) live virus neutralizing antibody titers of sera from each group of hamsters at Day 35. Viral loads of lungs (**D**) and nasal turbinates (**E**) determined by qRT-PCR. (**F**) Representative lung pathology scaled as mild, moderate or severe. Microscope images were taken at 100 × magnification. (G) Number of hamsters that displayed mild, moderate or severe lung pathology in each group. Each dot represents an individual hamster. Numbers on the top of each bar represent the value of geometric mean. Statistical analysis was performed using *t*-test with Welch’s correction. **P < 0.01; ****P < 0.0001.

We demonstrated the candidate vaccines are able to elicit robust immune responses in mice and NHPs. However, the ultimate goal of developing SARS-CoV-2 vaccine is to prevent viral infections and associated disease. Thus, we conducted a challenge study in golden Syrian hamsters (Fig. 5A), which is a good model to evaluate the protective efficacy of candidate vaccines [19], [20], [21], [22]. In this study, group of 10 hamsters were immunized intramuscularly with Alum/CpG adjuvanted SΔTM, Alum/CpG adjuvanted RBD or Alum/CpG alone on Day 0 and Day 21, and challenged with SARS-CoV-2 (10^5^ TCID_50_, *i.n.*). Blood was collected on Day 35 to prepare serum samples, and subjected to both ELISA and live virus-based neutralization assay (Fig. 5B and 5C). Results showed that RBD combined with Alum/CpG hardly induced binding antibody responses, whereas SΔTM combined with Alum/CpG induced high titers of binding antibodies (GMT = 62500) in golden Syrian hamsters. In addition, only Alum/CpG adjuvanted SΔTM group induced high nAb titers (GMT = 2195). For reference, the virus nAb titers of human convalescent sera were ≦100 using the same live virus-based neutralization assay performed by the same laboratory. Subsequently, hamsters were sacrificed seven days post infection (Day 49), virus loads of lungs and nasal turbinates were measured by quantitative real-time polymerase chain reaction (qRT-PCR) (copies/mL) (Fig. 5D and 5E). We observed a significant reduction in virus titers in lungs (3.00 log_10_) and nasal turbinates (0.99 log_10_) 7 days post intranasal virus infection in Alum/CpG adjuvanted SΔTM group. However, there were no significant differences in virus titers in lungs and nasal turbinates between Alum/CpG adjuvanted RBD group and Alum/CpG alone group.

We then evaluated the lung histopathology 7 days post intranasal virus infection by H&E staining. Hamsters in all groups showed varying degrees of lung inflammation with thickened alveolar septa. The lung pathology was scaled as mild, moderate or severe in this study (Fig. 5F). In Alum/CpG alone group, as a control, 4 of 6 hamsters showed severe lung inflammation; 2 of 6 hamsters showed moderate lung inflammation (Fig. 5G). In Alum/CpG adjuvanted SΔTM group, by comparison, 3 of 6 hamsters showed moderate lung inflammation; 3 of 6 hamsters showed mild lung inflammation (Fig. 5G). However, there were no significant reduction in lung pathology in Alum/CpG adjuvanted RBD group compared with Alum/CpG alone group, 4 hamsters developing severe lung inflammation and 2 hamsters developing moderate lung inflammation (Fig. 5G).

In summary, Alum/CpG adjuvanted SΔTM induced robust nAb responses. And the candidate vaccine can significantly reduce the viral loads in lungs and nasal turbinates after SARS-CoV-2 infection in hamsters. In addition, hamsters showed reduced lung pathology after immunized with Alum/CpG adjuvanted SΔTM. Taken together, these results demonstrated significant efficacy of Alum/CpG adjuvanted SΔTM in golden Syrian hamster challenge model.

### 2.5 Cross-reactivity with variants

**Fig. 6:**
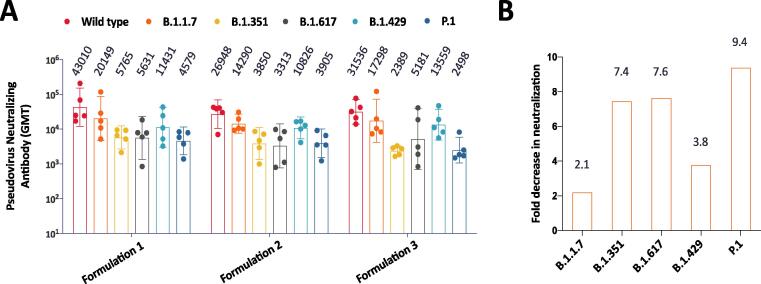
**Cross-reactivity with SARS-CoV-2 variants**. (**A**) Pseudovirus neutralizing antibody responses against both wildtype and variant SARS-CoV-2 as indicated. In this experiment, B.1.617 variant contains three mutants, including L452R, D614G and E484Q. Samples were Day 35 sera of NHPs vaccinated with formulation 1, formulation 2 or formulation 3 vaccines. (**B**) Fold decrease in neutralization relative to wild type SARS-CoV-2. Samples were Day 35 sera of NHPs vaccinated with formulation 1 vaccine.

As the COVID-19 pandemic goes viral, new variants emerged. Some of the new variants showed enhanced infectivity and/or immune escape [23]. This phenomenon is probably a challenge for the development of efficacious SARS-CoV-2 vaccines. Here we thawed sera collected 2 weeks after second immunization from NHPs and evaluated the cross-reactivity of vaccine-induced neutralizing antibodies against pseudoviruses that bear spike proteins from variants, including B.1.1.7, B.1.351, B.1.617, B.1.429 and P.1 strains (Fig. 6A). In the pseudovirus-based neutralization assay, results showed candidate vaccine-induced neutralizing antibodies cross-reacted with all these variants. However, the nAb titers against variants reduced in different degree compared with that of wild type (Fig. 6B). The mean fold decrease in neutralization relative to wild type was 2.1 folds for B.1.1.7, 7.4 folds for B.1.351, 7.6 folds for B.1.617, 3.8 folds for B.1.429, and 9.4 folds for P.1. Our data are in line with other studies that variants bearing E484K mutation in spike protein showed immune evasion in larger extent [24], [25], [26]. B.1.617 variant, first arising in India and currently circulating in many countries extensively, also showed significant decrease in neutralization relative to wild type. These results indicate SARS-CoV-2 viruses evolved and are still evolving to escape immune system.

## 3 Discussion

Currently, several SARS-CoV-2 vaccines have been approved and demonstrated to take into effect in controlling the COVID-19 pandemic. However, there is still a huge gap between the limited supply and the large population, especially in the developing countries. Subunit vaccines have been widely developed with a good record of safety and efficacy. Here we comprehensively evaluated the immunogenicity and protective efficacy of a recombinant spike protein vaccine adjuvanted with aluminium hydroxide and CpG 7909 in mouse, hamster and cynomolgus monkey models. Preclinical data demonstrated the vaccine candidate is promising, as it is able to induce potent nAb responses in all these animals and showed protective efficacy in hamsters after SARS-CoV-2 challenge. These data are in accordance with published data of similar vaccines developed by Clover Biopharmaceuticals and Medigen Vaccine Biologics Corporation [27], [28], [29], [30], in which CpG 1018 was used.

Both CpG 7909 and CpG 1018 are type B CpG ODN, which enhance vaccine-induced adaptive immune responses by stimulating human B cells and plasmacytoid dendritic cells. Previous studies on HBV vaccine development showed that when HBsAg adjuvanted with high dose CpG 1018 (3 mg/dose) elicited higher titers of antibody responses than adjuvanted with CpG 7909 [31], [32]. However, the immune persistence of CpG 7909 adjuvanted vaccine seems to be better than CpG 1018 adjuvanted [31], [32]. Thus, we chose CpG 7909 as the vaccine component. In preclinical study, our candidate vaccine induced comparable nAb responses with those adjuvanted with CpG 1018 at the peak. As there is no available data on longitudinal analysis of CpG 1018 adjuvanted SARS-CoV-2 vaccine-induced immune responses so far, we cannot confirm whether CpG 7909 adjuvanted SARS-CoV-2 vaccine could induce more durable immune responses than CpG 1018 adjuvanted. However, our candidate vaccines present excellent immune persistent in both mice and non-human primate models. Outstandingly, the decline of nAb titers from 12 weeks post second immunization to 24 weeks post second immunization is approximately 20% in our NHP study. In comparison, a RBD nanoparticle-based study adjuvanted with AS03 showed more than 2.5-fold decrease in nAb titers from 11 weeks post second immunization to 23 weeks post second immunization [33].

In the course of SARS-CoV-2 vaccine development, both Spike protein and RBD were utilized as vaccine immunogens and demonstrated to be immunogenic [27], [34], [35], [36], [37], [38]. In this study, we compared the immunogenicity between trimeric spike protein and monomer RBD. Trimeric spike protein induced more potent nAb responses than monomer RBD in both mouse and hamster models. Besides, only trimeric spike protein showed protective efficacy in hamster challenge model. These results are not unexpected as spike protein contains more neutralizing epitopes than RBD. In addition, spike protein as a trimer resembles its native structure, making it highly immunogenic. The unexpected is that RBD-induced nAbs were detected in mice but not golden Syrian hamsters. This phenomenon maybe explained by the difference of MHC class II molecule diversity and/or TCR repertoire between hamsters and mice, as the production of antibodies needs help from CD4^+^ T cells [39]. Our results are in line with a previous report [40], which showed RBD has limited immunogenicity in mice. In addition, the report found RBD is more immunogenic in non-human primates than in mice, analogous to the finding that RBD is more immunogenic in mice than golden Syrian hamsters in our study.

The emergence of new variants of SARS-CoV-2 is of great concern. Our results showed prototype SARS-CoV-2 vaccine can cross-neutralize variants. However, the nAb titers against P.1, B.1.351 and B.1.617 variants are significantly reduced compared with that against prototype. Other studies also reported the similar phenomenon [26]. These results suggest that vaccine-induced neutralizing antibodies may not be fully broadly neutralizing. Thus, it is necessary to develop a COVID-19 vaccine that is effective to prevent infections of major variants of SARS-CoV-2 continuously.

In summary, our vaccine candidate showed high immunogenicity and good protective efficacy in preclinical studies. These positive results support further clinical trials and paved the way for developing next generation vaccine against variants of SARS-CoV-2.

## 4 Methods

### 4.1 Cell culture

hACE2 expressing BHK-21 (BHK-21-hACE2) and Vero-E6 cells were provided from State Key Laboratory of Virology, Wuhan University. Vero-E6 cells were cultured in DMEM (Gibco, Cat. 11995–040) supplemented with 10% fetal bovine serum (Gibco, Cat. 10091–148) and 1% penicillin and streptomycin solution (BasalMedia, Cat. S110JV) at 37 °C with 5% CO_2_. BHK-21-hACE2 cells were cultured in the above medium supplemented with 1 μg/mL puromycin (Beyotime, Cat. ST511) under the same conditions.

### 4.2 Protein production and characterization

To express the prefusion spike protein, a gene encoding the ectodomain of SARS-CoV-2 S (GenBank: MN908947) with proline substitutions at residues 986 and 987, a “GGSG” substitution at the furin cleavage site (residues 682–685), a C-terminal T4 fibritin trimerization motif was synthesized and cloned into the mammalian expression vector. To express the RBD (RBD-SD1), residues 332–591 of SARS-CoV-2 spike protein were cloned into a different vector and a 6 × His tag was added to the N terminus. Both expression vectors were transfected into Chinese hamster ovary (CHO) cells. SΔTM was purified from clarified supernatant through low pH for preventative viral inactivation (VI), followed by three different chromatography steps to remove host cell DNA, host cell proteins, and any other impurities, and finally nanofiltration as a preventative viral removal (VR) step. While his-tagged RBD was purified using immobilized metal affinity chromatography (IMAC). SDS-PAGE and Size-Exclusion HPLC were run to check the purity of both proteins and the trimeric conformation of SΔTM.

Biolayer interferometry (BLI) assays were performed on a GatorPrime (GatorBio) instrument at 30 °C with shaking at 1,000 rpm. The Fc-tagged human ACE2 (Sino Biological, Cat. 10108-H02H) was immobilized to human Fc (HFC) Probes (Gator Bio, Cat. 160003) at 10 μg/mL in 10 × Kinetics Buffer. SΔTM and RBD were two-fold and three-fold serially diluted in 10 × kinetics buffer respectively prior to the measurement of association and dissociation rate. The data were baseline subtracted prior to fitting performed using a 1:1 binding model. Mean *k*_on_, *k*_off_ values were determined with a global fit applied to all data.

### 4.3 Mouse and nonhuman primate studies

Female specific pathogen free (SPF) BALB/c mice, 6–8 weeks of age, were obtained and housed in Shanghai SIPPR-BK Lab Animal Co., Ltd. All mouse experiments were approved by the Institutional Animal Care and Use Committee (IACUC) of Shanghai SIPPR-BK Lab Animal Co., Ltd. All mice were intramuscularly immunized on Day 0 and Day 21 respectively. Blood was collected at different timepoints to evaluate antibody responses via ELISA and neutralization assays. For some experiments, a subset of mice from groups was sacrificed to assess T cell responses by intracellular cytokine staining (ICS) and B cell memory by B cell ELISPOT, respectively.

Adult cynomolgus monkeys, weighting between 2.5 kg and 5.0 kg, were purchased from Guangxi Fangcheng Gang Spring Biological Technology Development Corporation Ltd. Animals were housed in United-Power Pharma Tech Co., LTD. (Guangxi) and all experiments were approved by the IACUC of United-Power Pharma Tech Co., LTD. Each group of 5 monkeys were intramuscularly immunized on Day 0 and Day 21. Blood was collected and sera were prepared on Day −7, Day 14, Day 35, Day 105 and Day 189 to detect antibody responses. In addition, blood was withdrawn on Day 35 and PBMCs were separated to evaluate vaccine-induced cellular immune responses by ICS and CBA.

### 4.4 Enzyme-linked immunosorbent assay (ELISA)

For the measurement of binding antibody titers, RBD or SΔTM proteins were diluted in phosphate-buffered saline (PBS) at 1 μg/mL and used to coat 96-well microplates (Corning, Cat. 9018) at 100 μL/well overnight at 2–8 °C. Plates were washed 5 times with PBST (PBS containing 0.05% Tween 80) and blocked with 5% milk in PBST at room temperature for 2 h. Sera were 2- or 5-fold serially diluted with 2% milk in PBST. Diluted sera were added into antigen coated plates and incubated at 37 °C for 1 h, then incubated with horseradish peroxidase (HRP) conjugated Goat anti-mouse IgG (Bio-Rad, Cat. 1706516) at 1:10000 dilution, Goat anti-hamster IgG (Invitrogen, Cat. PA1-28823) at 1:15000 dilution or Goat anti-monkey IgG (BETHYL, Cat. A140-102P) at 1:5000 dilution at 37 °C for 1 h. The plates were washed 5 times with PBST after each incubation. Then 100 μL TMB substrate system (SeraCare, Cat. 5120–0038) was added into each well and incubated for approximately 15 min. The color development was stopped by adding 50 μL 2 M sulfuric acid. Plates were read at 450 nm and 620 nm for absorbance by using a microplate reader (Molecular Devices, SpectraMax iD3). The endpoint titer is defined as the reciprocal of the highest serum dilution providing an OD_450nm-620nm_ value above 2.1-fold of the negative control. In situations where the OD_450nm-620nm_ value of the negative control is<0.05, regard it as 0.05.

For detecting IgG2a and IgG1 of sera from immunized mice, all procedures are the same as the above, except the following. After diluted sera were incubated with coated antigen, plates were washed 5 times with PBST and added anti-mouse IgG2a or IgG1 (Sigma, Cat. IS02) at 1:1000 dilution. Then, incubated another 1 h at room temperature before the reaction with HRP conjugated rabbit anti-goat IgG (Sigma, Cat. A5420) at 1:5000 dilution.

### 4.5 Pseudovirus-based neutralization assay

Pseudoviruses containing spike proteins from SARS-CoV-2 were prepared using a replication-deficient VSV-based rhabdoviral pseudotyping system expressing firefly 485 luciferase (VSV-dG-fluc), which was obtained from State Key Laboratory of Virology, Wuhan University. SARS-CoV-2 pseudovirus were generated according to a previously described protocol [41]. Briefly, Vero-E6 cells were transfected with the plasmids overexpressing SARS2-CoV-2 spike proteins (pCAGGS-SARS2-S-dc) using Lipofectamine 2000 reagent (Invitrogen, Cat. 11668–027). After 48 h, the transfected cells were transduced with VSV-dG-fluc reporter viruses for 1 h at 37 °C. Transduced cells were washed with PBS for 5 times and then replenished with fresh culture medium with anti-VSV monoclonal antibody (1:500 dilution) to neutralize the infectivity of the residual VSV-dG-fluc. SARS-CoV-2 pseudoviruses containing culture supernatants were collected 24 h later. The supernatants then subjected to centrifugation for 5 min at 2,000 rpm, and stored at − 80 °C. The titration of SARS-CoV-2 pseudovirus was performed by the 50% tissue culture infective dose method (TCID_50_) according to Reed-Muench.

For neutralization assay, 60 μL of heat inactivated sera were 2-fold serially diluted and added into 96-well plates (Corning, Cat. 3599), and 60 μL SARS-CoV-2 pseudoviruses diluted to contain 800 TCID_50_ were added into the plates. The mixture was incubated at 37 °C for 1 h, and then added to BHK-21-hACE2 cells in a 96-well white plate with clear bottom (Corning, Cat. 3610). Luciferase activity was measured 24 h later using luciferin-containing substrate (PerkinElmer, Cat. 6066769). The neutralizing titer was calculated by the dilution number of 50% inhibition condition. The neutralizing titer was calculated according to Reed-Muench method.

### 4.6 Live virus-based neutralization assay

The Live virus-based neutralization assay was performed by Biosafety Level 3 Laboratory, Shanghai Medical College, Fudan University and the Institute of Laboratory Animal Science, Chinese Academy of Medical Sciences (for hamster study only). All SARS-CoV-2 live virus-related experiments were conducted in the BSL-3 laboratory. Briefly, medium containing serum at varying dilutions in 96-well plates was pre-incubated with an equal volume of live SARS-CoV-2 solution diluted to contain 100 TCID_50_. After neutralization in a 37 °C for 1 h, the mixture was added to Vero E6 cells in a 96-well plate. After 3–5 days’ incubation at 37 °C, cytopathic effect (CPE) of each well was recorded under microscopes, and the neutralizing titer was calculated by the dilution number of 50% protective condition.

### 4.7 Intracellular cytokine staining (ICS) assay

Splenocytes and PBMCs were prepared from mice and NHPs respectively. Splenocytes or PBMCs (1 × 10^6^) were seeded into 96-well round bottom plates (Corning, Cat. 3799) and stimulated with 1% DMSO as negative control, 1 μg/mL/peptide of a peptide pool from SARS-CoV-2 spike protein (synthesized by GenScript, China) or 100 ng/mL PMA & 1 μg/mL Ionomycin as positive control. 1 μg/mL Brefeldin A (BD, Cat. 555029) was added to inhibit cytokine secretion. After incubation at 37 °C 5% CO_2_ for approximately 6 h, cells were harvested and stained with LIVE/DEAD Aqua and a panel of flow cytometry antibodies specific for cell surface markers: PB anti-mouse CD3 (BioLegend, Cat. 100214), FITC anti-mouse CD4 (BD, Cat. 553047) for mouse study or PB anti-human CD3 (BD, Cat. 558124), FITC anti-human CD4 (BD, Cat. 550628) for NHP study at 2–8 °C for 30 min. Following washing and permeabilization (BD, Cat. 554714), cells were further stained with flow cytometry antibody mixture: APC anti-mouse IFN-γ (BD, Cat. 554413), PE-Cy7 anti-mouse IL-2 (BD, Cat. 560538), PE anti-mouse TNF-α (BD, Cat. 554419) for mouse study or APC anti-human IFN-γ (BD, Cat. 554702), PE-Cy7 anti- human IL-2 (BD, Cat. 560707), PE anti- human TNF-α (BD, Cat. 557068) for NHP study at 2–8 °C for 30 min. The stained cells were analyzed by BD FACSCantoII flow cytometry.

### 4.8 Enzyme-linked immunospot (ELISPOT) assay

SΔTM-specific memory B cells were detected with Mouse IgG ELISpot kit (Mabtech, Cat. 3825-2A) according to the user manual. Briefly, the splenocytes were pre-incubated for 72 h in the presence of 1 μg/mL R848 and 10 ng/mL rmIL-2 to enrich memory B cells. Wells of PVDF-based membrane plates were coated with 150 μg/mL SΔTM protein overnight at 2–8 °C. Then plates were washed and blocked with RPMI 1640 containing 10% FBS. 3 × 10^5^ stimulated splenocytes were added into each well after removing secreted antibodies by two-round wash to incubated at 37 °C 5% CO_2_ incubator. Following a 24-hour incubation, the cells were removed, and the plates were washed 5 times with PBS and incubated with biotinylated anti-mouse IgG antibodies for 2 h. Next, plates were washed 5 times and incubated with alkaline phosphatase (AKP) conjugated streptavidin (BD, Cat.554065) at 1:2000 dilution for another 1 h. Then plates were washed 5 times with PBS and spots were visualized by adding 100 μL/well BCIP/NBT substrate solution (Thermo Fisher Scientific, Cat. 34042). Plates were left to dry in the dark. Spots were counted using ELISPOT reader (CTL, S6).

### 4.9 Cytometric bead array (CBA)

Cytokine secretion from stimulated PBMCs was determined by Non-Human Primate Th1/Th2 Cytokine kit (BD, Cat. 557800) according to manufacturer’s instruction manual, with minor modifications. Briefly, similar to ICS assay above, 1 × 10^6^ NHP PBMCs were seeded into 96-well plates and stimulated with 1% DMSO, peptide pool of spike protein or PMA & Ionomycin. After 18- hour incubation, cell culture was centrifugated and supernatant was harvested. Then 50 μL standards or supernatants from each sample were incubated with 20 μL mixture of Capture Beads and 25 μL Detection Reagent at room temperature for 3 h, protected from light. The samples were washed with 1 mL Wash Buffer. Finally, samples were resuspended in 150 μL Wash Buffer and analyzed samples with BD FACSCantoII flow cytometry.

### 4.10 Hamster challenge study

SPF golden Syrian hamsters, 6–10 weeks of age, were obtained from Beijing Vital River Laboratory Animal Technology Co., Ltd. and housed in Institute of Laboratory Animal Science, Chinese Academy of Medical Sciences (CAMS). All hamster experiments were approved by the IACUC of the Institute of CAMS. Hamsters were intramuscularly immunized twice on Day 0 and Day 21 respectively. On Day 35, blood from all hamsters was collected to detect antibody responses. On Day 42, all hamsters were challenged intranasally with 10^5^ TCID_50_ SARS-CoV-2, isolate of SARS-CoV-2/WH-09/human/2020/CHN. On Day 49, a subset of hamsters in each group was euthanized for detecting viral loads of lungs and nasal turbinates by qRT-PCR and evaluating lung histopathology by hematoxylin and eosin (H&E) staining. Both qRT-PCR and H&E staining were performed using published protocols [19], described briefly in the following paragraphs.

To detect tissue viral loads by qRT-PCR, total RNA was extracted from lung and turbinate tissue homogenates by the RNeasy Mini Kit (Qiagen) according to manufacturer instructions. Reverse transcription was completed by the PrimerScript RT Reagent Kit (TaKaRa) and qRT-PCR was conducted utilizing the PowerUp SYBR Green Master Mix Kit (Applied biosystems). The forward and reverse primers targeting SARS-CoV-2 envelope protein (E) gene for qRT-PCR were 5′- TCGTTTCGGAAGAGACAGGT −3′ and 5′- GCGCAGTAAGGATGGCTAGT −3′, respectively. qRT-PCR was conducted by an ABI 3730 DNA sequencer (Applied Biosystems) under the following reaction conditions: 1) 50 °C for 2 min; 2) 95 °C for 2 min; 3) 40 cycles of 95 °C for 10 s, and 60 °C for 30 s; 4) 60 °C for 1 min; and 5) 95 °C for 45 s.

To evaluate lung histopathology by H&E staining, lung tissues from hamsters were fixed by 10% buffered formalin and processed for paraffin embedding. Paraffin blocks were cut into 5-μm sections and stained with hematoxylin and eosin. Lung pathology, including overall lesion extent, pneumocyte hyperplasia, and inflammatory infiltrates, was assessed and classified into 3 types: mild, moderate and severe lung inflammation.

### 4.11 Statistical analysis

All statistical analyses were performed using GraphPad Prism. Antibody titers and viral loads were log_10_ transformed prior to statistical analysis. Differences among two groups were analyzed using Mann-Whitney test. Values of p < 0.05 are considered statistically significant.

**Funding**

This work was partly supported by the Bill & Melinda Gates Foundation (BMGF) (Investment ID: INV-006445).

**Author contributions**

Liu, G. oversaw and conceptualized the project, participated in study design and data analysis as well as revision of the manuscript. Zhou, C. and Jiang, Y. led the team efforts to conduct immunogenicity/efficacy studies, and production and analysis of vaccine candidates, respectively. An, J. and Song, Y. designed vaccine immunogens, and led the team efforts to develop purification and formulation process. Zhou, C., Liu, H., Hu, D., Jiang, Y., Zhou, L., and Hu, Z. coordinated the experiments. Liu, H., Li, J., Zhang, L., Huang, C., Zhang, C., Yang, Y., Zhu, Q., Wang, D., Liu, Y., Miao, C., and Cao, X. performed all experiments, except the hamster challenge study, the live virus-based neutralization assays, and the antigenicity study. Liu, J., Yu, P., Zhu, H., and Bao, L. coordinated and conducted the hamster challenge study. Xie, Y., Gu, C., and Zhu, Y. coordinated and conducted the live virus-based neutralization assays. Xu, J. and Ding, L. coordinated and conducted the ELISA to measure the antigenicity of immunogens. Liu, H., Li, J., and An, J. wrote the manuscript. Zhang, C., Zhou, C., and Fan, J. revised the manuscript.

## Declaration of Competing Interest

The authors declare that they have no known competing financial interests or personal relationships that could have appeared to influence the work reported in this paper.
